# Relational Needs in Grief Scale: Development and Psychometric Validation

**DOI:** 10.3390/bs16030348

**Published:** 2026-02-28

**Authors:** Alexandra Coelho, Sara Albuquerque, David Dias Neto, Miguel Barbosa

**Affiliations:** 1 APPsyCI—Applied Psychology Research Center Capabilities & Inclusion, ISPA—Instituto Universitário, 1149-041 Lisbon, Portugal; dneto@ispa.pt; 2 PsyLab, Instituto de Saúde Ambiental (ISAMB-FMUL), Faculdade de Medicina, Universidade de Lisboa, 1649-028 Lisboa, Portugal; 3 InLuto—Associação Portuguesa de Cuidados Integrados no Luto, 1050-094 Lisboa, Portugal; 4 HEI-Lab: Digital Human-Environment Interaction Labs, Lusófona University, 1749-024 Lisboa, Portugal; sara.albuquerque@ulusofona.pt; 5 Centro de Investigação em Ciência Psicológica (CICPSI), Faculdade de Psicologia, Universidade de Lisboa, 1649-013 Lisboa, Portugal; miguel.barbosa@psicologia.ulisboa.pt; 6 Instituto de Saúde Ambiental (ISAMB-FMUL), Faculdade de Medicina, Universidade de Lisboa, 1649-028 Lisboa, Portugal

**Keywords:** relational needs, grief, scale development, psychometric validation

## Abstract

The disruption of attachment bonds through bereavement often leads to unfulfilled relational needs, emphasizing the importance of evaluating these processes systematically. Based on Erskine and Payàs’s conceptualization of relational needs, the present study aims to develop and psychometrically validate the Relational Needs in Grief Scale (RNGS). Data from 354 bereaved participants in an online cross-sectional survey were collected to investigate the instrument’s factorial structure, reliability, convergent validity, discriminant validity, and incremental validity. Results from Exploratory Factor Analysis identified two factors: “Need for Protection and Validity” and “Need for Mutuality”. Confirmatory Factor Analysis confirmed the scale’s two-dimensional nature. Stepwise elimination of underperforming items led to substantial improvements in model fit. The Need for Protection and Validation was positively associated with attachment-related anxiety and negatively with avoidance, and it significantly predicted prolonged grief symptoms. The final 11-item total scale and subscales yielded high internal consistency reliabilities (Cronbach’s α: 0.81–0.94, McDonald’s ω: 0.81–0.95) and satisfactory convergent, discriminant, and incremental validity. The RNGS constitutes a novel and psychometrically valid tool for both research and clinical practice, enabling the systematic assessment of the relational needs profile and informing the development of tailored interventions.

## 1. Introduction

A significant loss, particularly under traumatic circumstances, can profoundly disrupt an individual’s attachment security and generate intense feelings of isolation and loneliness (Neria & Litz, 2004). This attachment disruption precipitates a cascade of unmet relational needs as the bereaved attempt to reestablish a sense of safety, seek validation for their grief, and navigate their changed identity following the death of a significant other (Bottomley et al., 2022, 2025; Cacciatore et al., 2021; Mikulincer et al., 2003; Mikulincer & Shaver, 2022; Neimeyer et al., 2006). In attempting to fulfil their inherent desire for connection and understanding, bereaved individuals may experience changes in the availability, quality, or responsiveness of their social environment, which may exacerbate interpersonal strain and complicate the process of adjusting to the loss (Bottomley et al., 2022; Oliver, 1999).

The absence or inadequacy of social support is a well-established risk factor for the development of prolonged grief disorder (Harrison et al., 2022), a condition marked by persistent yearning, intense emotional pain, and significant functional impairment that endures beyond culturally expected grieving periods (Prigerson et al., 2021). Moreover, individuals with insecure attachment styles may be particularly vulnerable to prolonged grief reactions, as they often relied on the deceased as a primary source of emotional security. Such dependency can severely impair their ability to seek alternative relational bonds or communicate their relational needs clearly following the loss (Harrison et al., 2022). For instance, widowed older adults often experience heightened emotional loneliness and social isolation, as the deceased partner had served as their central attachment figure. Instead of reaching out for alternative sources of support, they may withdraw from social networks, reinforcing feelings of abandonment and intensifying their grief-related reactions (W. Stroebe et al., 2005). These interpersonal challenges can lead to emotional numbing and social avoidance, further isolating bereaved individuals and impeding their ability to reestablish secure attachments after the loss (Jerga et al., 2011).

Longitudinal research shows that, unlike social loneliness, emotional loneliness—a distressing response to the unmet need for intimacy and emotional connection that cannot be compensated by mere social contact (Breen & O’Connor, 2011)—is strongly associated with prolonged grief symptoms and may even be intensified by the ongoing experience of grief (Vedder et al., 2021). Despite the evident impact of such relational difficulties on bereavement outcomes, the perceived support needs of bereaved individuals have received little systematic attention (Breen, 2021; Cacciatore et al., 2021). The relational needs model, originally developed by Erskine (1998) and later adapted to the bereavement context by Payàs (2010), offers a useful framework for conceptualizing the fundamental relational needs that become particularly salient in the grieving process.

### 1.1. Conceptualization of Relational Needs

Relational needs, as originally conceptualized by Erskine (1998, 2011) and (Erskine et al., 2013), refer to specific interpersonal desires and expectations that are inherent to humans functioning and can only be satisfied within relational contexts. These needs reflect the innate human longing for connection that spans the life cycle, from early childhood through older adulthood. Erskine’s model (Erskine, 1998; Erskine et al., 2013) delineates eight core relational needs: (1) the need for security; (2) the need to feel validated, affirmed, and significant within a relationship; (3) the need to be accepted by a stable, dependable, and protective other; (4) the need for confirmation of personal experience; (5) the need for self-definition; (6) the need to have an impact on the other; (7) the need to have the other person initiate; and (8) the need to express love. These needs are considered universal and inherent to everyday relational life, although they often remain outside of conscious awareness. Nevertheless, when repeatedly unsatisfied, people often experience a sense of insecurity, which can lead to insecure attachment styles that may be pervasive in future relationships (Erskine, 2011; Maçkalı et al., 2025). As Erskine (1998) suggests, the therapeutic setting is, like no other, the space to recognize and respond appropriately to relational needs in a genuinely healing encounter that reestablishes both internal and external contact.

Building on Erskine’s conceptualization of relational needs (Erskine, 1998), Payàs (2010) outlined the fundamental bereavement-specific needs that most people experience in their social environment after a significant loss. These include (1) the need to be listened to and believed in one’s full grief story; (2) the need to be protected and permitted to express emotions; (3) the need to be validated in one’s individual way of grieving; (4) the need to engage in supportive and reciprocal relationships; (5) the need to define oneself in one’s unique way of living through grief; (6) the need to feel that one’s grief has an impact on others; (7) the need to be in relationships where the other takes the initiative; and (8) the need to express love and vulnerability.

This perspective provides a framework that translates Erskine’s universal relational dynamics into concrete expressions of relational needs in the context of grief. However, it is grounded mainly in theoretical reflection and clinical observation, and thus still lacks systematic empirical validation. Table 1 presents a consolidated overview of relational needs relevant to bereavement, including their conceptual definitions, roles in shaping grief experiences, and major theoretical implications. Through this synthesis, we advance an integrated account informed by the broader literature on social support, attachment processes, and interpersonal dynamics in the context of grief.

### 1.2. Evaluation of Relational Needs

The concept of relational needs has been empirically operationalized in the development of the Relational Needs Satisfaction Scale (RNSS) by Žvelc et al. (2020), designed primarily for psychotherapy and counselling contexts. This instrument moves beyond broad measures of relationship satisfaction by focusing on specific interpersonal needs that contribute to a deeper sense of relational fulfilment. Although the original theoretical model proposed eight components, exploratory factor analysis identified a more stable five-factor structure, accounting for 47.47% of the variance. These factors include: (1) support and protection, reflecting the need to feel accepted and safe with a dependable other; (2) having an impact on others; (3) shared experience, in line with the mutuality need; (4) the need for authenticity, which encompasses the desire to be fully oneself in the presence of others and includes aspects such as security, validation, and self-definition; and, finally, (5) having initiative from others. While the RNSS provides a nuanced framework for assessing relational fulfilment in everyday contexts, it does not specifically capture the unique relational needs that arise in the context of grief and loss.

Existing measures of relational dynamics in grief primarily focus on perceived social support (Li et al., 2022) and the quality of the bereaved–decedent relationship (Bottomley et al., 2019; Bottomley & Neimeyer, 2021). Additionally, the Inventory of Social Expectations in Bereavement (ISEB) was recently developed by Wanza et al. (2025) to assess grief-specific social expectations, particularly negative beliefs about how others might respond to expressions of grief. This instrument captures anticipatory cognitions that may contribute to feelings of loneliness and social withdrawal, such as fears of being misunderstood, judged, or perceived as burdensome. While innovative in targeting the socio-cognitive correlates of bereavement, the ISEB assesses expectations rather than actual relational experiences or needs. As such, it does not directly evaluate the degree to which relational needs—as intrinsic human relational motivations—are satisfied in the context of grief.

More broadly, a substantial body of instruments has been developed to assess grief-related symptomatology and associated constructs. Measures such as the PG-13 (Prigerson et al., 2009, 2021) and related diagnostic tools have primarily focused on the severity and clinical features of prolonged grief disorder. Other widely used instruments assess theoretically related dimensions frequently examined in bereavement research, including attachment insecurity (e.g., the Experiences in Close Relationships; Fraley et al., 2011), loneliness, and perceived social support (Boelen et al., 2003; Hughes et al., 2004). These tools have been validated across both community and clinical bereaved populations and have substantially advanced the empirical understanding of grief-related distress. In addition, several measures have been developed or adapted for specific populations and loss contexts. For example, the Marwit–Meuser Caregiver Grief Inventory and its short form have been validated in caregivers of people with dementia, a group frequently exposed to prolonged and ambiguous relational loss (Sánchez-Alcón et al., 2024). Similar psychometric efforts have addressed parental bereavement (e.g., Arnold et al., 2005) and traumatic loss (e.g., Boelen et al., 2003), reflecting methodological rigor and contextual sensitivity in grief assessment. However, despite their robustness, most existing instruments are primarily symptom-focused, outcome-oriented, or context-specific. They assess levels of distress, attachment insecurity, or perceived support but do not directly operationalize relational needs as intrinsic interpersonal motivations that may become activated or frustrated in the aftermath of loss. Consequently, a conceptual and measurement gap remains regarding the systematic assessment of relational needs as a distinct dimension of the bereavement experience.

This conceptual gap underscores the need for assessment tools that move beyond perceived or expected support and instead explore how bereaved individuals experience the fulfilment or frustration of their actual relational needs during the grieving process. Therefore, the present study aims to develop and psychometrically assess the Relational Needs in Grief Scale (RNGS), a measure designed for use with bereaved adults in the general population, encompassing both community and clinical contexts and a broad range of loss circumstances (e.g., illness-related death, sudden loss, anticipatory grief). Rather than targeting a specific diagnostic group or type of loss, the RNGS seeks to capture core relational processes that cut across grief trajectories, while acknowledging that their expression and salience may vary as a function of sociodemographic, relational, and contextual factors (e.g., Maciejewski et al., 2022; Mikulincer & Shaver, 2022). By capturing these needs, the RNGS has the potential to illuminate their associations with loneliness, prolonged grief symptoms, and difficulties in forming new bonds, thereby advancing theoretical understanding of relational processes in bereavement. Additionally, it offers a clinically useful tool for identifying specific domains of relational vulnerability and informing tailored psychosocial interventions. To our knowledge, the RNGS is the first instrument to directly assess relational needs specific to bereavement, filling a notable gap in existing measures and opening new avenues for empirical investigation.

## 2. Materials and Methods

### 2.1. Scale Development

The instrument was developed in three phases. First, an initial pool of 46 items was created to reflect the relational needs, based on the models of Erskine (1998, 2011), Erskine et al. (2013) and Payàs (2010). Additionally, we were inspired by the Relational Needs Satisfaction Scale (RNSS) and insights from clinical work with bereaved individuals. The aim was to capture the specific experiences and relational needs of bereaved individuals in everyday life. Items were evaluated for clarity and intelligibility by the authors, and those deemed ambiguous, unclear, or redundant were removed. This process resulted in two items representing each of the eight relational needs (Table 2), yielding a final set of 16 items, for a parsimonious scale. Then, in the second phase, the items were randomly ordered and submitted to a Delphi study with an expert panel to assess item wording, relevance, and construct validity (the procedures and results of this study will be reported in a separate manuscript).

In the third phase, the 16-item scale was administered to a sample of bereaved participants, who were instructed: “Please read each item carefully and reflect on how it applies to your personal experience of bereavement. I need someone to…”. Responses were provided on a five-point Likert scale ranging from 1 (“nothing”) to 5 (“extremely”). Simultaneously, additional instruments were administered to collect sociodemographic and loss-related information for sample characterization, as well as to assess convergent validity. The total sample was then randomly split into two subsamples: one used to examine the factor structure through Exploratory Factor Analysis (EFA), and the other to perform Confirmatory Factor Analysis (CFA) to evaluate model fit, reliability, and convergent validity.

### 2.2. Participants

The determination of sample sizes for the factor analyses followed the recommendations of R. C. MacCallum et al. (1999), who emphasize that requirements for adequate sample size in factor analysis depend on the communalities of the variables, the overdetermination of factors, and the magnitude of factor loadings. For the EFA, a sample of *n* = 165 was used, which meets the suggested criteria for models with moderate communalities (0.40–0.70) and well-determined factors (at least four indicators per factor) under moderate loading sizes. For the CFA, a separate sample of *n* = 189 was employed. Simulation results reported by R. C. MacCallum et al. (1999) indicate that a sample size of this magnitude is adequate for CFA models of moderate complexity, with similar communalities and factor structures, ensuring stable estimation and acceptable power for model fit assessment. The use of independent samples for EFA and CFA aligns with best practices to avoid overfitting and to provide unbiased cross-validation of the measurement model (Worthington & Whittaker, 2006). Participants were recruited using non-probabilistic convenience sampling complemented by snowball procedures. Inclusion criteria included: (a) age ≥ 18 years; (b) experience of the death of a significant person; and (c) ability to complete the questionnaire in Portuguese. Exclusion criteria comprised failure to provide informed consent and questionnaires with excessive missing data.

### 2.3. Instruments

*Sociodemographic and bereavement-related questionnaire.* This includes questions referring to sociodemographic information (age, gender, educational level, marital status, occupation, monthly net individual income) and the loss experience, namely the relationship to the deceased (Spouse/Partner, Parent, Child, Sibling, Grandparent, Uncle/Aunt, Parent-in-law, Friend, Other), the deceased’s age, if they cohabited (Yes/No), the cause of death (Oncological disease, Neurodegenerative disease, Organ failure, Accident, Suicide, Homicide, Other), and date of death (converted in in time since death, months).

*Experiences in Close Relationships—Relationship Structures Questionnaire* (*ECR-RS*; Fraley et al., 2011). It is a self-report instrument designed to assess adult attachment across different relational contexts, namely mother, father, romantic partner, and best friend. The Portuguese adaptation was developed by Moreira et al. (2015), maintaining the original bidimensional structure of attachment for each relational domain: avoidance (6 items) and anxiety (3 items). Items are rated on a 7-point Likert scale, from 1 (strongly disagree) to 7 (strongly agree), with higher scores indicating greater attachment insecurity. The CFA in this study supported the two-factor structure of the Portuguese version, and internal consistency coefficients were satisfactory, ranging from α = 0.87 to 0.92 for avoidance and from α = 0.75 to 0.91 for anxiety, depending on the relational context.

*Prolonged Grief Disorder PG-13—Revised Version* (*PG-13-R*; Prigerson et al., 2021). This instrument comprises 13 items assessing symptoms of prolonged grief disorder, according to DSM-5-RT (American Psychiatric Association, 2022). It includes one question concerning the time elapsed since the loss, ten statements rated on a five-point Likert scale from 1 (“not at all”) to 5 (“overwhelmingly”) that evaluate the intensity of prolonged grief symptoms, and two additional dichotomous items (yes/no) that focus on the loss event and functional impairment. The symptom subscale yields scores between 10 and 50, with values of 30 or above suggesting the presence of prolonged grief disorder. Cronbach’s alpha values range from 0.83 to 0.93 (Prigerson et al., 2021). In the present sample, the internal consistency was also considered very good (α = 0.86).

The ECR-RS and the PG-13-R were selected to evaluate construct validity based on theoretically proximal domains. Attachment orientations were included due to their conceptual relevance to relational security and dependency processes, and prolonged grief symptoms were assessed to examine associations with clinically relevant grief outcomes and incremental validity. As the RNGS assesses relational needs (i.e., desired relational responses) rather than emotional states or perceived support, validation prioritized adjacent constructs in this initial phase.

### 2.4. Procedures

This study is part of a larger research project that was submitted to and approved by the Ethics Committee of Lusófona University (reference: CEDIC-2024-4-19). Recruitment of participants took place between December and May 2025 and was conducted entirely online. The study was advertised across widely used social media platforms, including Facebook, Instagram, Twitter, and LinkedIn, as well as relevant online communities and interest groups related to bereavement and mental health. Posts inviting participation included a brief description of the study’s purpose, eligibility criteria, and estimated completion time, along with the direct survey link via Qualtrics. This recruitment method was chosen to maximize reach and accessibility, allowing participation from individuals in various geographical locations. Participants who accessed the link received full information about the study and provided informed consent before completing the questionnaire. They were informed that their participation was voluntary, responses would be kept anonymous and confidential, and they could withdraw at any stage. At the end of the questionnaire, information about national mental health services contacts was provided in case the loss-related questions caused distress. To enhance sample heterogeneity and mitigate potential biases associated with online non-probabilistic sampling, recruitment messages were standardized and disseminated across diverse digital contexts, including general social media networks and bereavement-focused communities.

### 2.5. Data Analyses

The IBM SPSS (version 30.0) statistical package was used for the descriptive and inferential analyses of the variables. A total of 244 participants were excluded due to missing data. Descriptive statistics were calculated for sociodemographic (age, gender, marital status, education level, occupation, and monthly net individual income) and bereavement-related characteristics (relationship with the deceased, age of the deceased, cohabitation with the deceased, cause of death and time since death), using measures of central tendency and dispersion, as well as frequency distributions for categorical variables.

For factor analysis, the full sample was randomly split into two approximately equal subsamples, using SPSS. The first subsample was used to conduct the EFA, and the second subsample was used for the CFA. Prior to conducting the main analyses, a series of preliminary comparisons between the two samples was performed using chi-square tests for categorical variables and independent samples *t*-tests for continuous variables (age and time since loss) to assess group equivalence. The EFA and CFA were performed using JASP 0.19.3 software.

The suitability of the dataset for factor analysis was assessed using the Kaiser–Meyer–Olkin (KMO) measure of sampling adequacy and Bartlett’s test of sphericity. The KMO statistic was computed both overall and for each item, with values above 0.80 considered indicative of meritorious sampling adequacy. Bartlett’s test was used to assess whether the correlation matrix differed significantly from the identity matrix (*p* < 0.05). Analyses were carried out using a polychoric correlation matrix with maximum-likelihood extraction and promax (oblique) rotation. Factor retention was guided by multiple criteria: Parallel analyses, the Kaiser criterion (eigenvalues > 1), inspection of the scree plot for points of inflexion, and the proportion of variance explained by each solution. Both one- and two-factor solutions were examined for statistical adequacy and conceptual interpretability. For each solution, we inspected factor loadings, communalities, and total variance explained. Following the recommendations for EFA, items with factor loadings above 0.40 and communalities above 0.40 were retained in the final solution (Hair, 2019). CFA was conducted to test the hypothesized factor structure, using maximum likelihood estimation. Model fit was assessed with multiple indices, adopting the following cut-off values as indicative of good fit: CFI and TLI ≥ 0.95, RMSEA ≤ 0.08, SRMR ≤ 0.08, and GFI ≥ 0.90; McDonald’s fit index (MFI) values ≥ 0.85 were considered satisfactory (Brown, 2015).

Descriptive statistics, including means, standard deviations, skewness, and kurtosis, were computed for the RNGS total and subscale scores to characterize central tendency, variability, and distributional properties. The internal consistency of the RNGS scale was assessed using Cronbach’s alpha coefficient, with values above 0.70 considered acceptable for research purposes and values above 0.80 indicative of good reliability (Nunnally & Bernstein, 1994). McDonald’s omega (ω) was also computed as a complementary reliability estimate. Omega values above 0.70 were interpreted as indicating adequate internal consistency (Dunn et al., 2014). Omega is considered a more robust measure when factor loadings are heterogeneous, as it does not require equal item covariances. The combined use of alpha and omega provided a more comprehensive evaluation of the scale’s internal reliability. Inter-item and item-total correlations were computed to evaluate the strength and consistency of associations among items. Inter-scale Pearson correlation coefficients were also calculated to examine the relationships between RNGS factors.

Convergent validity was assessed using the Average Variance Extracted (AVE) and composite reliability (CR) indices, both calculated from the standardized factor loadings of the measurement model. AVE values of 0.50 or higher were considered indicative of adequate convergent validity, meaning that, on average, the construct explains at least 50% of the variance in its indicators (Fornell & Larcker, 1981). Composite reliability values above 0.70 were interpreted as evidence of satisfactory internal consistency. The combined evaluation of AVE and CR provided a robust assessment of the extent to which the items reflect their respective latent constructs. Discriminant validity was evaluated using the Fornell–Larcker criterion, which requires that the square root of the AVE for each construct be greater than its correlations with any other construct in the model. Meeting this condition indicates that a construct shares more variance with its indicators than with other constructs, supporting its empirical distinctiveness within the measurement model. Additionally, associations with theoretically related constructs were examined by calculating Pearson correlation coefficients between the RNGS subscales and the attachment dimensions (Avoidance and Anxiety). A linear regression analysis was conducted with the RNGS subscale scores as predictors of Prolonged Grief Disorder symptom severity to assess the incremental validity of the measure.

Group differences in RNGS subscale mean scores were examined according to sociodemographic and bereavement-related characteristics, using independent-samples *t*-tests for variables with two categories and one-way ANOVA for those with more than two. To ensure adequate group sizes, some categories were combined. Gender was coded as male or female; age was categorized as 18–29, 30–44, 45–59, and 60–80 years; marital status was classified as single, married/common-law union, or separated/divorced/widowed. Educational level was categorized into two groups: those without a university degree and those with a university degree/higher. Occupational status was categorized into four groups: students/student workers, employed, unemployed/retired/on sick leave/or other. Monthly net income was classified as less than €1200 or €1200 or more. Kinship with the deceased was categorized as either first-degree relatives (parents, children, siblings) or other relationships (grandparents, friends, and others). The age of the deceased was divided into four categories: 0–17, 18–44, 45–64, and 65 years or older. Cohabitation with the deceased was recorded as yes or no, and cause of death as natural causes (oncological, degenerative, organ failure) or non-natural causes (accident, suicide, homicide). Time since death (in months) was categorized as ≤6, 7–12, 13–60, or more than 60. Effect sizes for *t*-tests were calculated using Cohen’s *d* (0.20 = small, 0.50 = medium, 0.80 = large) and for ANOVA using eta-squared (η^2^) (0.01 = small, 0.06 = medium, 0.14 = large). Post hoc ANOVA comparisons were conducted with Tukey’s HSD test

## 3. Results

### 3.1. Sociodemographic and Bereavement-Related Characteristics

Table 3 presents the sociodemographic and bereavement-related characteristics of the participants. The total sample comprised 354 participants, with a mean age of 33.99 years (SD = 15.26, range = 18–80). Most identified as women (74.9%), were single (65.5%), and had attained higher education, with nearly half holding a licentiate degree and over one-fifth a master’s degree (20.3%). In terms of occupational status, the majority were employed (44.1%) or students (32.2%). Regarding income, about one-third reported no personal income (31.4%), followed by those earning between €850 and €1200 (17.2%) and between €1200 and €1800 (16.1%).

Concerning the bereavement-related variables, the most common losses were of a grandparent (46.3%) or parent (27.4%), with the deceased having a mean age of 67.49 years (SD = 21.92). The majority did not cohabit with the deceased (77.7%). The most frequently reported causes of death were organ failure (33.3%) and oncological disease (31.4%). On average, the loss had occurred 86.15 months prior to participation (SD = 92.77).

Chi-square tests and independent samples *t*-tests were performed to compare the two independent samples (*n* = 189 vs. *n* = 165) across sociodemographic and bereavement-related variables. No statistically significant differences were found for gender, χ^2^(4) = 6.48, *p* = 0.166; age, *t*(350) = −1.25, *p* = 0.212, Cohen’s *d* = −0.13; nationality, *χ*^2^(1) = 0.56, *p* = 0.455 (Fisher’s exact *p* = 0.610); marital status, *χ*^2^(3) = 2.76, *p* = 0.430; educational level, *χ*^2^(8) = 5.08, *p* = 0.749; occupational status, *χ*^2^(6) = 7.83, *p* = 0.251; monthly net individual income, *χ*^2^(9) = 5.90, *p* = 0.750; kinship with the deceased, *χ*^2^(8) = 5.57, *p* = 0.696, age of the deceased, t(335) = −1.55, *p* = *0*.122, Cohen’s *d* = −0.169; cohabitation with the deceased, *χ*^2^(1) = 6.48, *p* = 0.964; cause of death, *χ*^2^(6) = 4.76, *p* = 0.575 and time since loss (in months), *t*(350) = −1.09, *p* = 0.277, Cohen’s *d* = −0.12. These results indicate that the two samples did not differ significantly on any of the demographic or bereavement-related variables examined, suggesting that they are comparable for subsequent analyses.

### 3.2. Exploratory Factor Analysis

Data adequacy for EFA was confirmed (KMO = 0.944; Bartlett’s test of sphericity was significant, *χ*^2^(120) = 6683.945, *p* < 0.001). Parallel analysis supported a one-factor solution, and a maximum-likelihood model (promax rotation) accounted for 66.7% of the variance. Standardised loadings on the common factor ranged from 0.584 to 0.927, with communalities from 0.341 to 0.860. Global model fit was *χ*^2^(104) = 1141.685, *p* < 0.001. Given that the eigenvalue greater than one criterion suggested retaining two factors, we also estimated a two-factor solution, which improved fit and explained variance. After promax rotation, Factor 1 and Factor 2 accounted for 54.5% and 18.9% of the variance, respectively; communalities ranged from 0.564 to 0.868. Collectively, the two-factor model reduced *χ*^2^ from 1141.685 (*df* = 104) to 771.411 (*df* = 89) and increased explained variance from 66.7% to 73.4%, indicating improved data fit. Factor 1 aggregated items 1, 3, 4, 5, 7, 8, 9, 10, 12, 13, 14, and 16, and was called “Protection and Validation”; Factor 2 included items 2, 6, 11, and 15, and was labelled “Mutuality” (Table 4). These results reflect the initial 16-item solution; subsequent CFA-based refinement yielded the final 11-item version reported below.

### 3.3. Confirmatory Factor Analysis

A CFA was conducted to evaluate the fit of the hypothesised two-factor model. The initial model demonstrated suboptimal fit indices, *χ*^2^(103) = 347.258, *p* < 0.001, CFI = 0.914, TLI = 0.900, RMSEA = 0.112 (90% CI [0.099, 0.125]), and SRMR = 0.051. Stepwise elimination of underperforming items—specifically, items 5, 9 and 14 due to low standardised loadings, and items 1 and 2 due to high residual variances—led to substantial enhancements in model fit, *χ*^2^(43) = 91.643, *p* < 0.001, CFI = 0.969, TLI = 0.961, RMSEA = 0.077 (90% CI [0.055, 0.099]), SRMR = 0.038. However, the values still fell short of the recommended thresholds.

Examination of the modification indices suggested that model fit could be further improved by permitting residual covariances between items 4 and 13 (covariance = −0.208) and between items 13 and 7 (covariance = −0.155), resulting in an acceptable model fit, *χ*^2^(41) = 67.291, *p* = 0.006, CFI = 0.983, TLI = 0.978, RMSEA = 0.058 (90% CI [0.031, 0.083]), SRMR = 0.036. Although chi-square fit indices should be nonsignificant (*p* > 0.05), a significant chi-square is common with large samples and data from Likert scales.

**Figure 1:**
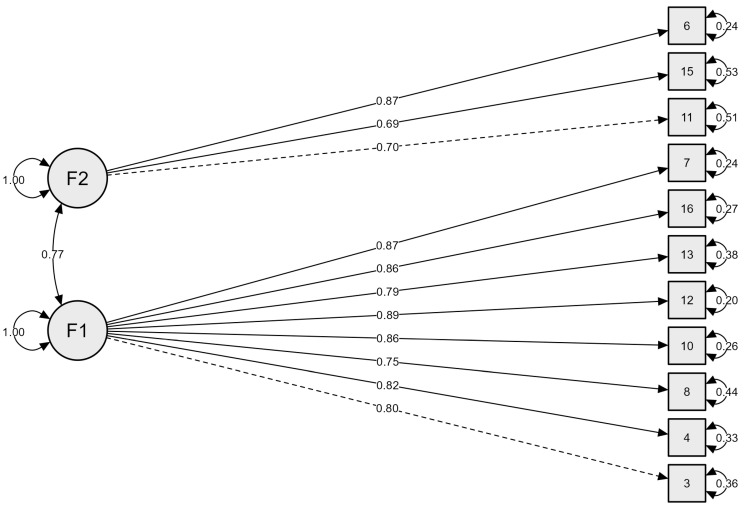
FCA Model plot.

As shown in Figure 1, the final two-factor CFA model demonstrated strong standardised loadings for most items, with residual variances ranging from 0.20 to 0.53 and a latent factor correlation of 0.77. Compared with the initial model, goodness-of-fit index (GFI) increased from 0.803 to 0.938, and McDonald’s fit index (MFI) improved from 0.524 to 0.933, indicating a substantial enhancement in overall model performance. The Parsimony-Adjusted Normed Fit Index (PNFI) was 0.715, exceeding the commonly suggested minimum value of 0.50 for an acceptable parsimony-adjusted fit, indicating that the model achieves a good balance between fit quality and simplicity.

Although the final model includes an unequal number of items across factors, this asymmetry appears to reflect the broader conceptual scope of Protection and Validation compared to the more specific construct of Mutuality. Item reduction aimed solely at numerical symmetry was not pursued to preserve conceptual coverage and internal consistency. The final 11-item version of the scale is provided as Supplementary Material (Portuguese and English versions).

### 3.4. Descriptives and Reliability

Following item elimination (Items 1, 2, 5, 9, and 14), the remaining items were renumbered sequentially from 1 to 11. Descriptive statistics (Table 5) revealed that the RNGS total score had a mean of 2.99 (*SD* = 1.08), positioning it near the midpoint of the 1–5 response scale. Among the subscales, the Need for Protection and Validation exhibited the highest mean score (*M* = 3.25, *SD* = 1.19), whereas the Need for Mutuality had a mean score of 2.25 (*SD* = 1.03). Within the Need for Protection and Validation dimension, Item 11 (original item 16: “Allow me to share my true feelings without fear of criticism”) presented the highest mean score (*M* = 3.41), while Item 1 (original item 3: “Allow me to show my vulnerability”) showed the lowest (*M* = 2.91). For the Need for Mutuality dimension, the highest mean score was observed for Item 3 (original item 6: “Show that one is touched by my experience”; *M* = 2.49), whereas the lowest corresponded to Item 10 (original item 15: “Be moved by my pain”; *M* = 1.94). Skewness values ranged from −0.54 to 1.14 and kurtosis values from −1.23 to 0.88, all within widely accepted thresholds (|Sk| < 2; |Ku| < 3), indicating approximately normal distributions of scores.

Cronbach’s alpha (α) was 0.940 for Factor 1, 0.807 for Factor 2 and 0.942 for the total scale, all exceeding the 0.70 benchmark for acceptable reliability. McDonald’s omega (ω) values were 0.955 for Factor 1 and 0.808 for Factor 2, and 0.951 for the total scale, indicating similar and satisfactory levels of internal consistency. These results, together with the CR values (0.876 and 0.803), reinforce the scale’s reliability across both dimensions. Inter-item correlations ranged from 0.552 (RNGS_7) to 0.872 (RNGS_6), surpassing the recommended 0.30 threshold (Table 3). The two Factors were strongly and positively correlated (*r* = 0.651, *p* < 0.001).

### 3.5. Convergent and Discriminant Validity

Convergent validity was supported, as the average variance extracted (AVE) was 0.502 for Factor 1 and 0.506 for Factor 2, both above the 0.50 cut-off. Discriminant validity was also confirmed: for each factor, the square root of AVE (0.709 for Factor 1 and 0.711 for Factor 2) exceeded the inter-factor correlation (0.543), and the heterotrait–monotrait ratio of correlations (HTMT) was 0.622, remaining well below the 0.85 threshold.

### 3.6. Incremental Validity

Hierarchical multiple regression using the continuous PG-13 total score (*N* = 150) as the criterion indicated that the Need for Protection and Validation significantly predicted prolonged grief symptoms in the first step, accounting for 3.5% of the variance (*R*^2^ = 0.035, *β* = 0.186, *p* = 0.023). Adding the need for mutuality in the second step did not yield a significant increase in explained variance (ΔR^2^ < 0.001, *p* = 0.823), and its coefficient was not significant (β = −0.023, *p* = 0.823).

### 3.7. Associations with Related Constructs

Associations with theoretically related constructs were further examined through Pearson correlation analyses with attachment dimensions. The Need for Protection and Validation was negatively associated with Avoidance (*r* = −0.193, *p* < 0.001) and positively associated with Anxiety (*r* = 0.446, *p* < 0.001). The Need of Mutuality showed no significant association with Avoidance (*r* = −0.103, *p* = 0.054) but correlated positively with Anxiety (*r* = 0.237, *p* < 0.001).

### 3.8. Differences by Sociodemographic and Bereavement-Related Characteristics

Table 6 presents group differences in sociodemographic characteristics. Significant gender differences emerged, with women scoring higher than men on both Need for Protection and Validation and Need for Mutuality. Age group differences were found for Need for Protection and Validation, with participants aged 18–29 reporting higher scores than those aged 30–44. No age differences emerged for Mutuality. Marital status was significant for both dimensions, with single participants scoring the highest, and the divorced/widowed the lowest. Occupation also showed differences. For Need for Protection and Validation, students/student workers scored higher than employed participants. For Mutuality, students/student workers and unemployed/retired/on sick leave/other scored higher than employed. No differences were found for educational level. Income effects were significant: participants earning less than €1200/month reported higher Need for Protection and Validation and Mutuality. Bereavement-related characteristics showed no differences by kinship (*p*s ≥ 0.063), deceased’s age (*p*s ≥ 0.449), cohabitation (*p*s ≥ 0.576), cause of death (*p*s ≥ 0.561), or time since death (*p*s ≥ 0.318).

## 4. Discussion

The results of this study provide strong psychometric evidence supporting the Relational Needs in Grief Scale (RNGS) as a concise and theoretically grounded instrument for assessing relational needs in the context of grief. The final 11-item scale includes two factors: the Need for Protection and Validation (Factor 1), which comprises eight items addressing interpersonal needs for security, affirmation, and support, and the Need for Mutuality (Factor 2), which contains three items that reflect the bereaved’s request for mutual exchange of emotional experiences. The total scale and its subscales demonstrated strong internal consistency and satisfactory convergent, discriminant, and incremental validity. Together, these findings indicate that the RNGS captures central interpersonal processes involved in grief adjustment and can be meaningfully applied in both research and clinical contexts.

### 4.1. Relational Needs in Grief: Convergence and Divergence with Previous Research

From a theoretical perspective, the distinction between Protection and Validation and Mutuality reflects complementary but functionally distinct aspects of relational functioning in bereavement. The Protection and Validation dimension highlights the need for emotional safety, reassurance, and recognition through being listened to and having one’s inner experience acknowledged. This pattern closely aligns with attachment theory’s conceptualization of the secure base and safe haven, whereby responsive relationships support emotion regulation during periods of heightened vulnerability (Erskine, 2011; Shaver & Mikulincer, 2002).

In contrast, Mutuality captures the bidirectional nature of relational engagement, including the ability to give and receive emotional support. This dimension reflects ongoing interpersonal exchanges in which bereaved individuals are not only recipients of care but also active contributors to relational bonds, thereby fostering interdependence and regulating grief over time (Ratcliffe & Byrne, 2021). Together, these dimensions offer a nuanced conceptual framework in which Protection and Validation provides containment and affirmation during vulnerability, while Mutuality promotes agency and connectedness through active relational exchange and shared meaning-making through reciprocal relational exchange.

Importantly, the prominence of Protection and Validation helps explain why the RNGS yielded a two-factor structure, diverging from broader relational frameworks such as Erskine’s eight relational needs (Erskine, 1998, 2011) or the five-factor structure identified in the Relational Needs Satisfaction Scale (Žvelc et al., 2020). In the context of bereavement, several relational needs that are differentiated in everyday life—such as security, validation, self-definition, and having an impact—may converge into a broader experiential need to feel emotionally safe and understood. Similar patterns of construct convergence have been observed in other emotionally intense life events, where heightened salience leads to stronger interrelations among psychological dimensions that are otherwise more distinct (Bleidorn et al., 2018; Jayawickreme & Blackie, 2014; Luhmann et al., 2012). These findings suggest that context-specific adaptations of relational needs measures may yield more parsimonious structures without diminishing theoretical relevance.

### 4.2. Attachment Orientations and Relational Needs in Grief

The associations between relational needs and attachment orientations further support the theoretical coherence of the RNGS. The Need for Protection and Validation was associated with higher attachment-related anxiety and lower avoidance, suggesting that individuals presenting these needs tend to be more preoccupied with relational security and less prone to distancing themselves from others emotionally. This relational profile, characterized by a strong desire for reassurance and a readiness to seek and accept support, contributes to explaining why Protection and Validation emerged as a significant predictor of prolonged grief symptoms. When such needs remain unmet, the bereaved may experience persistent feelings of insecurity and a heightened dependence on others, which can hinder the adaptive processing of loss (Mikulincer & Shaver, 2022; M. K. Shear, 2015). In these cases, the absence of sufficient emotional affirmation and a sense of safety may perpetuate rumination and intensify yearning for the deceased, sustaining distress over time (Boelen & Lenferink, 2020).

Moreover, the tendency to remain highly attuned to relational cues, typical of high attachment anxiety and low avoidance, can exacerbate sensitivity to perceived unavailability or lack of responsiveness from others, further complicating grief adjustment (Fraley & Bonanno, 2004). These findings converge with previous research highlighting the role of attachment insecurity in maladaptive grief trajectories and underscore the importance of relational containment in bereavement adjustment.

In contrast, the Need for Mutuality was positively associated with attachment anxiety but not with avoidance, suggesting that individuals with higher attachment anxiety may have a greater need for mutual sharing of emotional experiences as a way to seek reassurance and maintain closeness (Mikulincer & Shaver, 2022). On the other hand, the absence of a link with avoidance indicates that mutuality may be less about overcoming relational distance and more about fostering a sense of connection through bidirectional emotional exchange. In the context of bereavement, this pattern implies that mutuality plays a more selective role in adjustment—supporting emotional sharing and mutual understanding—but is less central than protection and validation in regulating distress and facilitating the integration of the loss (M. S. Stroebe & Schut, 2001; Field et al., 2013). This aligns with research showing that, while mutuality can buffer stress in close relationships, its role in grief adaptation may be secondary to the provision of emotional security and validation during periods of heightened vulnerability (Landolt et al., 2023; Eisma et al., 2023).

### 4.3. Psychometric Refinement and Item Reduction

Following identification of the two-factor structure, the measurement model was refined through confirmatory factor analysis to optimise model fit and ensure that all retained items contributed meaningfully to the latent constructs. Despite EFA indicating an acceptable factor structure and initially retaining all items, CFA applies stricter, theory-driven constraints and evaluates model fit through multiple indices simultaneously (Schreiber et al., 2006). Under these conditions, several items that appeared adequate in the EFA presented low standardised loadings or high residual variances in the CFA, leading to their removal.

Items reflecting trust, general validation, or legitimacy of pain (Items 5, 9, and 14) showed weaker alignment with the latent constructs, likely due to heterogeneous interpretation or contextual dependency (e.g., variation in reference relationship; Boateng et al., 2018; Worthington & Whittaker, 2006). Likewise, items with high residual variance (Items 1 and 2) may have reflected redundancy with related indicators or strong situational conditionality, particularly where shared loss experiences were assumed. Their exclusion improved factorial purity and model fit, thereby strengthening the theoretical coherence of the RNGS by retaining indicators most consistently aligned with relational needs in bereavement (Howard, 2016; Ziegler & Hagemann, 2015).

### 4.4. Sociodemographic Patterns and Stability of Relational Needs

The Need for Protection and Validation emerged as the most strongly endorsed relational need, confirming the importance of emotional safety, affirmation, and support in the bereavement context. The prominence of this need was especially evident among women, younger adults, single individuals, students or student workers, and those with lower income. These patterns are consistent with previous findings linking these groups to higher perceived interpersonal support needs during stressful life transitions (Benkel et al., 2009; Burke & Neimeyer, 2013). In contrast, relational needs did not vary concerning bereavement-related characteristics such as kinship to the deceased, time since death, cohabitation, age of the deceased or cause of death, which may indicate a relatively stable interpersonal orientation that endures across different grief situations.

Although the theoretical perspective suggests temporal variation in relational needs, our study did not identify significant differences. This null finding may reflect both methodological constraints, particularly the small number of participants in the acute phase, and the possibility that relational needs serve stable adaptive functions across bereavement trajectories. This stability aligns with evidence that core relational patterns, influenced by attachment history and personality traits, tend to remain consistent even after significant loss (Fraley & Shaver, 1999; Lobb et al., 2010).

### 4.5. Clinical Implications

The RNGS offers clinically valuable insights by identifying and quantifying specific relational needs that may influence adaptation to bereavement. By distinguishing between the Need for Protection and Validation and the Need for Mutuality, the scale enables practitioners to tailor interventions according to the relational needs profile of the bereaved. For instance, individuals scoring high on Protection and Validation may benefit most from interventions that prioritise emotional safety, consistent affirmation, and the reduction of relational insecurity, such as attachment-informed grief therapy (Kosminsky & Jordan, 2016; M. K. Shear, 2015). Conversely, those with elevated Mutuality needs may respond well to group-based or peer-support approaches that emphasise shared emotional experiences and mutual help (Morrissey et al., 2024). Screening with the RNGS can thus guide clinicians in selecting interventions that align with each bereaved person’s relational priorities, potentially improving therapeutic engagement and outcomes.

Beyond treatment selection, the RNGS can also serve as a monitoring instrument to identify changes in relational needs throughout grief therapy. Persistent high levels of unmet Protection and Validation needs, particularly when accompanied by elevated prolonged grief symptoms, may indicate difficulties in emotional integration of the loss, signalling the need for targeted intervention. Furthermore, the identification of relational needs as predictors of prolonged grief underscores their potential as clinical risk markers, supporting early identification and preventive strategies for individuals at heightened risk of maladaptive bereavement trajectories (F. Maccallum & Bryant, 2013; Boelen & Lenferink, 2020). The therapeutic relationship holds the potential to offer a reparative and transformative experience, grounded in deep attunement and responsive relational support (Sayar & Hjeltnes, 2021). Hence, the RNGS not only advances research into the interpersonal dimensions of grief but also enhances the precision and responsiveness of bereavement support.

### 4.6. Limitations and Suggestions for Future Studies

A central limitation of the present study concerns the use of a broad general-population sample without stratification by specific grief contexts or participant characteristics. Although this approach was intentionally adopted to support the initial development and psychometric validation of the Relational Needs in Grief Scale (RNGS) as a transdiagnostic instrument, it inevitably introduced substantial heterogeneity in grief experiences and relational needs. Such variability may have increased dispersion in the data and limited the identification of more fine-grained or subgroup-specific patterns. In addition, the absence of stratification by relevant characteristics—such as age, type of loss, social context, or participant role—constrains the generalizability of the findings to specific bereaved populations and reduces the precision of conclusions regarding how relational needs may manifest across different grief trajectories.

In addition to these considerations, several methodological limitations should be acknowledged when interpreting the findings. First, its cross-sectional design precludes causal inferences regarding the relationships between relational needs and grief outcomes. The observed associations may reflect bidirectional or context-dependent influences that can only be clarified through longitudinal designs, which would enable the examination of how relational needs evolve over time and across different phases of bereavement. Second, although the sample size met the general requirements for factor analysis, it was relatively modest for a full-scale psychometric validation, which limited the statistical power to detect more subtle patterns and potentially contributed to sample-specific factor structures. Additionally, we did not restrict the time frame, as loss was not used as an inclusion criterion, which may have introduced additional variability and potentially diluted effects specific to particular time frames (e.g., acute vs. longer-term grief).

Furthermore, the sample was not fully representative of the broader bereaved population: specific subgroups—particularly widowed individuals, older adults, people with lower educational levels, and those in the acute phase of grief—were underrepresented. This limits the generalisability of the results to more diverse bereavement experiences. In addition, only five participants in the sample had experienced the loss of a child. Parental bereavement—particularly perinatal loss—is widely recognised as one of the most severe and socially complex forms of grief, often involving stigma, disenfranchisement, and challenging interactions with healthcare professionals. These contextual dynamics may activate additional or distinct relational needs that were not fully captured in a sample largely composed of grandparent and parent losses. Therefore, the generalisability of the RNGS to bereaved parents remains limited and should be specifically examined in future validation studies (e.g., Dahò, 2020).

Finally, the unequal distribution of items across the two factors suggests that Protection and Validation may capture a broader constellation of needs, whereas Mutuality is represented more narrowly. This discrepancy highlights both the centrality of Protection and Validation in grief adjustment and the need for further refinement of the RNGS to ensure balanced representation of relational dimensions.

Future research should address these limitations by employing larger and more heterogeneous samples, with deliberate oversampling of underrepresented groups to ensure greater diversity in demographic and bereavement-related circumstances. Longitudinal studies are particularly warranted to assess the stability and predictive value of relational needs across different grief trajectories, including the transition from acute to integrated grief. It would also be essential to examine the RNGS in cross-cultural contexts, as relational expectations and coping strategies during bereavement may vary across cultural backgrounds. Moreover, testing the scale in clinical populations, such as individuals seeking professional grief support or those diagnosed with Prolonged Grief Disorder, could provide valuable evidence of its sensitivity to clinically relevant variations in relational needs. Future research should seek to replicate and extend these findings using more comprehensive validation batteries. In particular, studies may broaden the validation framework of the RNGS by incorporating complementary outcome-based measures (e.g., loneliness or perceived social support), thereby strengthening evidence of convergent and discriminant validity.

## 5. Conclusions

The findings of this study support the Relational Needs in Grief Scale (RNGS) as a psychometrically sound measure with a clear two-factor structure encompassing Protection and Validation, and Mutuality. These dimensions reflect core relational processes that are relevant to how individuals experience and regulate grief within their relational environments. By assessing relational needs as interpersonal motivations rather than as symptoms or perceived support, the RNGS contributes to a clearer conceptualization of bereavement as a relational process. The observed associations with grief-related outcomes indicate that unmet relational needs represent a meaningful component of grief adjustment and warrant consideration in both research and practice. As a transdiagnostic instrument, the RNGS is applicable to bereaved adults across diverse loss contexts in community and clinical settings. The scale offers a concise and clinically relevant tool for identifying relational vulnerabilities in grief and provides a foundation for future studies examining relational processes across different bereavement trajectories.

## Figures and Tables

**Table 1:** Conceptualization of Relational Needs in the Bereavement Context.

Relational Need	Conceptual Definition	Relevance in Bereavement	Key Implications
Security/Protection	Attachment relationships provide physical and emotional safety, predictability, and the freedom to express vulnerability without fear of rejection (Bowlby, 1980; Erskine, 1998).	The absence of a protective attachment figure (e.g., a spouse) has been associated with heightened anxiety, depressive symptoms, and adverse physical health outcomes (Mikulincer & Shaver, 2022).	When this need is unmet, proximity-seeking behaviours, typically expressed as yearning for the deceased, remain unfulfilled and may hinder grief adaptation (Payàs, 2010; Eisma & Lenferink, 2023).
Being Listened to/Feeling Valued	Feeling heard, validated, and understood by another person who non-judgmentally acknowledges one’s inner experience (thoughts, feelings, and perceptions) is a core way of feeling valued (Payàs, 2010).	In grief, the bereaved often carry intense, confusing, or socially challenging emotions and need to repeatedly narrate aspects of the loss as part of integrating the traumatic experience (Payàs, 2010).	High-quality listening promotes felt relatedness and a deeper sense of connection, thereby easing the psychological burden associated with experiences of rejection that resemble the relational disruption of bereavement (Itzchakov et al., 2023).
Acceptance/Validation	Acceptance refers to the ongoing need to be cared about, respected, needed, and affirmed by others, particularly by a strong, stable, and reliable person who offers protection, encouragement, and direction (Erskine et al., 2013).	Bereavement can profoundly challenge the need for acceptance and approval, especially when the deceased was the primary source of support; their absence may elicit feelings of alienation from others whose lives appear to continue unchanged (Ratcliffe & Byrne, 2021).	Validating their emotional expression and individual coping style normalizes grief and reduces discomfort, reinforcing a sense of fundamental adequacy despite societal pressures (Payàs, 2010; Larsen et al., 2025).
Mutuality	Mutuality denotes the desire for shared emotional engagement and reciprocity in relationships, with a sense of being genuinely understood, often by others who have undergone similar experiences (Erskine et al., 2013).	In the context of grief, mutuality can emerge through connections with others who have experienced comparable losses, providing a context in which emotions, experiences, and coping strategies are shared and affirmed (Bentum & Lynn Gall, 2017; Payàs, 2010).	Shared understanding normalizes their emotional responses and reduces isolation, particularly within support groups or empathic social networks; this process is especially salient in stigmatized losses (Buyukcan-Tetik et al., 2022; Hybholt et al., 2022; Morrissey et al., 2024; Uno, 2025; Bottomley et al., 2025).
Self-Definition	Self-definition reflects the need to express one’s uniqueness and to feel that others genuinely recognized and respect it, including the freedom to assert personal values, preferences, and individuality without fear of rejection or ridicule (Erskine, 2011).	This need becomes particularly salient when expressions of grief are minimized or socially disapproved, and as the bereaved attempt to redefine themselves in the absence of the deceased, especially when the relationship was central to their identity (Payàs, 2010; Wehrman, 2023).	Supportive relationships that honour the bereaved’s evolving self-concept without imposing expectations of stability or continuity are crucial for integrating the loss into a coherent personal narrative (Jiang et al., 2025; Benkel et al., 2024).
Having an Impact on Others	Having an impact on others corresponds to a fundamental human need to matter—that is, to feel that one’s thoughts, emotions, and actions have meaningful effects on others (Erskine, 1998, Erskine et al., 2013).	Bereaved individuals may experience themselves as invisible or emotionally insignificant, which can constitute a significant source of traumatization, particularly when social reactions minimize or dismiss their pain (Payàs, 2010; Bottomley et al., 2022).	When others genuinely respond—by remembering the deceased, acknowledging the bereaved’s pain, or engaging in shared rituals—it can help restore a sense of agency and relational efficacy (Benkel et al., 2024; Cesur-Soysal & Arı, 2024; Maciejewski et al., 2022).
Initiative of Others	The need for the initiative of others refers to the desire to be actively sought out, remembered, and included in a reciprocal relationship, without continually initiating contact or requesting attention (Erskine et al., 2013).	Bereaved individuals frequently experience emotional loneliness and may withdraw from social contact; those with prolonged grief disorder may show notably diminished responsiveness and difficulty adapting to relational changes (Niino et al., 2025; Harrison et al., 2022).	The bereaved are often emotionally unavailable and require that the other person assume initiative in the relationship; such proactive, compassionate engagement reduces social withdrawal and exemplifies a public-health–oriented approach to bereavement support (Payàs, 2010; Tognela et al., 2025; Aoun, 2020).
Express Love	The need to express love refers to the intention to offer affection, care, esteem, and emotional investment, with the expectation that this will be welcomed, reciprocated, or at least respected (Erskine et al., 2013).	In bereavement, the target of love is no longer physically available, and individuals may yearn for love that will never again be received, or that was never fully experienced in the relationship; the absence of space to express love may inhibit vulnerability and intimacy and foster fear of forming new attachments (Payàs, 2010; K. Shear et al., 2007).	When individuals are prevented from expressing love, their emotional well-being may be significantly compromised; thus, facilitating opportunities to express enduring love for the deceased (e.g., through memorialization or shared reminiscence) and gently supporting the re-engagement of affectionate bonds in existing or new relationships is crucial (Floyd, 2014; Harrison et al., 2022; Erbiçer et al., 2023).

**Table 2:** Correspondence between RNGS’s items and Erskine’s/Payà’s Relational Needs Models.

RNGS Items	Erskine’s Relational Needs	Payà’s Relational Needs
7. Make me feel safe and stable in my relationships. 9. Make me feel that I can truly trust him/her.	Security	Need to be protected and permitted to express emotions
5. Listen to and accept what I have to say. 1. Take seriously what I am going through.	Valuing	Need to be listened to and believed in one’s full grief story
14. Recognise my pain as legitimate and valid. 12. Do not minimise or judge when I express my feelings.	Acceptance	Need to be validated in one’s individual way of grieving
2. Share with me feelings of grief similar to mine. 11. Share that they have been through the same grief experience.	Mutuality	Need to engage in supportive and reciprocal relationships
13. Respect my particular way of grieving. 16. Allow me to share my true feelings without fear of criticism.	Self-definition	Need to define oneself in one’s unique way of living through grief
15. Be moved by my pain. 6. Show that my experience touches them.	Making an impact	Need to feel that one’s grief has an impact on others
4. Help me without me having to ask. 10. Remember to ask me how I am feeling.	Having the other initiate	Need to be in relationships where the other takes the initiative
3. Allow me to show my vulnerability. 8. Allow me to express how much I loved the person I lost.	Expressing love	Need to express love and vulnerability

**Table 3:** Sociodemographic and Bereavement-related Characteristics (N = 354).

Variable	*n*	%
**Age**, *M* (*S.D.*)	33.99 (15.26)	
Range	18–80	
**Gender**		
Woman	265	74.9
Man	82	23.2
Non-binary person	3	0.8
Prefer not to answer	1	0.3
Other	3	0.8
**Marital status**		
Single	232	65.5
Married/Common-law union	82	23.2
Separated/Divorced	34	9.6
Widowed	6	1.7
**Educational level**		
<4th grade	1	0.3
6th grade	1	0.3
9th grade	4	1.1
12th grade	70	19.8
Technical course	19	5.4
Bachelor’s degree	8	2.3
Licentiate degree	169	47.7
Master’s degree	72	20.3
Doctorate	10	2.8
**Occupation**		
Student	114	32.2
Working student	49	13.8
Employed	156	44.1
Unemployed	12	3.4
Retired	14	4.0
On medical leave	2	0.6
Other	7	2.0
**Monthly net individual income**		
No personal income	111	31.4
<€600	28	7.9
€600–€850	27	7.6
€850–€1200	61	17.2
€1200–€1800	57	16.1
€1800–€2500	40	11.3
€2500–€4000	5	1.4
€4000–€6000	6	1.7
>€6000	2	0.6
Prefer not to answer/Do not know	17	4.8
**Relationship to the deceased**		
Spouse/Partner	8	2.3
Parent	97	27.4
Child	5	1.4
Sibling	12	3.4
Grandparent	164	46.3
Uncle/Aunt	19	5.4
Parent-in-law	1	0.3
Friend	28	7.9
Other	20	5.6
**Age of deceased,** *M* (*S.D*.)	67.49 (21.92)	
Range	7–99	
**Cohabitation with the deceased**		
No	275	77.7
Yes	79	22.3
**Cause of death**		
Oncological disease	111	31.4
Neurodegenerative disease	33	9.3
Organ failure	118	33.3
Accident	21	5.9
Suicide	11	3.1
Homicide	4	1.1
Other	56	15.8
**Time since death, Months** *M* (*S.D*.)	86.15 (92.77)	
Range	1–563	

**Table 4:** Factor loadings and communalities for the RNGS scale items.

Item	Factor 1	Factor 2	Communality
9. Make me feel that I can truly trust him/her.	0.996	−0.098	0.858
16. Allow me to share my true feelings without fear of criticism.	0.974	−0.039	0.894
12. Do not minimize or judge when I express my feelings.	0.940	0.002	0.887
14. Recognize my pain as legitimate and valid.	0.877	0.051	0.837
13. Respect my particular way of grieving.	0.874	−0.005	0.758
7. Make me feel safe and stable in my relationships.	0.847	0.059	0.795
5. Listen to and accept what I have to say.	0.836	0.082	0.807
3. Allow me to show my vulnerability.	0.821	−0.066	0.598
8. Allow me to express how much I loved the person I lost.	0.782	0.113	0.754
10. Remember to ask me how I am feeling.	0.725	0.186	0.758
4. Help me without me having to ask.	0.586	0.245	0.615
1. Take seriously what I am going through.	0.562	0.267	0.608
6. Show that one is touched by my experience.	0.147	0.772	0.783
11. Share that one has been through the same grief experience.	−0.102	0.853	0.610
15. Be moved by my pain.	0.032	0.777	0.641
2. Share with me feelings of grief similar to mine.	0.030	0.716	0.546

**Table 5:** Descriptives of the Relational Needs in Grief Scale (N = 354).

	Min	Max	Mean	SD	Skewness	Kurtosis
Protection and Validation	1	5	3.24	1.18	−0.50	−0.93
Item 1 (3)	1	5	2.91	1.356	−0.082	−1.231
Item 2 (4)	1	5	2.95	1.317	−0.113	−1.141
Item 4 (7)	1	5	3.37	1.362	−0.543	−0.907
Item 5 (8)	1	5	3.18	1.366	−0.315	−1.131
Item 6 (10)	1	5	3.22	1.41	−0.285	−1.202
Item 8 (12)	1	5	3.37	1.436	−0.479	−1.109
Item 9 (13)	1	5	3.34	1.395	−0.446	−1.09
Item 11 (16)	1	5	3.41	1.432	−0.519	−1.074
Mutuality	1	5	2.24	1.02	0.6	−0.30
Item 3 (6)	1	5	2.49	1.226	0.353	−0.867
Item 7 (11)	1	5	2.31	1.253	0.544	−0.79
Item 10 (15)	1	5	1.94	1.151	1.136	0.466
RNGS_Total	1	5	2.99	1.07	−0.39	0.88

Note. Items were renumbered in the final version of the scale after the exclusion of Items 1, 2, 5, 9 and 14. Original numbering is shown in parentheses.

**Table 6:** Differences in Relational Needs across sociodemographic characteristics.

		Need for Protection and Validation					Need for Mutuality				
Variable	Category	*M*	Test	*p*	Effect Size	Post Hoc	*M*	Test	*p*	Effect Size	Post Hoc
Gender	Women	3.39	*t*(345) = 3.75	<0.001	*d* = 0.47	-	2.77	*t*(345) = 3.31	0.001	*d* = 0.42	-
	Men	2.85					2.34				
Age group	18–29	3.53	*F*(3,348) = 11.14	<0.001	η^2^ = 0.088	18–29 > others	2.77	*F*(3,348) = 2.25	0.083	η^2^ = 0.019	n.s.
	30–44	3.15					2.61				
	45–59	2.88					2.55				
	60–80	2.47					2.29				
Marital status	Single	3.46	*F*(2,351) = 12.28	<0.001	η^2^ = 0.065	Single > others	2.77	*F*(2,351) = 3.87	0.022	η^2^ = 0.022	Single > Div/Wid
	Married	2.94					2.48				
	Div./Wid.	2.66					2.39				
Occupation	Students	3.61	*F*(2,351) = 16.28	<0.001	η^2^ = 0.085	Students > Employed	2.85	*F*(2,351) = 7.53	<0.001	η^2^ = 0.041	Students/Other > Employed
	Employed	2.89					2.42				
	Other	3.17					2.82				
Education	≥Univ.	3.25	*t*(352) = 0.10	0.923	*d* = 0.01	n.s.	2.69	*t*(352) = 0.97	0.334	*d* = 0.12	n.s.
	<Univ.	3.24					2.57				
Income	<€1200	3.56	*t*(352) = −4.83	<0.001	*d* = −0.51	-	2.86	*t*(352) = 3.40	<0.001	*d* = −0.36	-
	≥€1200	2.97					2.48				

Note. *M* = mean; *t* = independent-samples *t* test; *F* = one-way ANOVA; η^2^ = eta squared; *d* = Cohen’s *d*; Div./Wid. = divorced/widowed; Univ. = university; n.s. = non-significant; n.a. = not applicable. Post hoc tests were conducted using Tukey HSD.

## Data Availability

The data are available upon request to the corresponding author.

## Supplementary Materials

The following supporting information can be downloaded at: https://www.mdpi.com/article/10.3390/bs16030348/s1. The final validated version of the Relational Needs in Grief Scale (Portuguese and English versions) is available online.
