# Supplementary material for: Relational Needs in Grief Scale: Development and Psychometric Validation
Source: Behav Sci (Basel). 2026 Feb 28;16(3):348. doi: 10.3390/bs16030348 (PMC13024076; doi:10.3390/bs16030348)
Supplement: Supplementary file 1 [file behavsci-16-00348-s001.zip › behavsci-4089871-supplementary.pdf]

Relational Needs in Grief Scale
(Eng. Version)

This questionnaire aims to explore the relational needs of bereaved individuals in relation to their close support network. Please read each item carefully and reflect on how it applies to your personal experience of grief, namely the extent to which the following needs are met in your relationships.

I need someone who...

	Nothing (1)	A Little (2)	Modera-tely (3)	Much (4)	Extre-mely (5)
1. Allow me to show my vulnerability.					
2. Help me without me having to ask.					
3. Show that one is touched by my experience.					
4. Make me feel safe and stable in my relationships.					
5. Allow me to express how much I loved the person I lost.					
6. Remember to ask me how I am feeling.					
7. Share that one has been through the same grief experience.					
8. Do not minimize or judge when I express my feelings.					
9. Respect my particular way of grieving.					
10. Be moved by my pain.					
11. Allow me to share my true feelings without fear of criticism.					

Factor 1 – Protection and Validation: Items 1, 2, 4, 5, 6, 8, 9, 11
Factor 2 – Mutuality: Items 3, 7, 10

Escala das Necessidades Relacionais no Luto
(Port. Version)

Este questionário tem como objetivo explorar as necessidades relacionais de pessoas em luto face à sua rede de suporte próxima. Leia cada item atentamente e reflita sobre como este se aplica à sua experiência pessoal de luto, nomeadamente até que ponto as seguintes necessidades são satisfeitas nas suas relações.

Tenho necessidade que alguém que...

	Nada (1)	Um Pouco (2)	Moderadamente (3)	Muita (4)	Extremamente (5)
1. Me permita mostrar a minha vulnerabilidade					
2. Me ajude sem eu precisar de pedir					
3. Se mostre tocado/a pela minha experiência					
4. Me faça sentir segurança e estabilidade nas minhas relações					
5. Me permita expressar o quanto gostava da pessoa que perdi					
6. Se lembre de me perguntar como me sinto					
7. Partilhe que já passou pela mesma experiência de luto					
8. Não minimize ou julgue quando expresso os meus sentimentos					
9. Me respeite na minha forma particular de fazer o luto					
10. Se comova com a minha dor					
11. Me permita partilhar os meus verdadeiros sentimentos, sem receio da crítica					

Fator 1 – Proteção e Validação: Itens 1, 2, 4, 5, 6, 8, 9, 11
Fator 2 – Reciprocidade: Itens 3, 7, 10
